# Polymer Microfluidics: Simple, Low-Cost Fabrication Process Bridging Academic Lab Research to Commercialized Production

**DOI:** 10.3390/mi7120225

**Published:** 2016-12-10

**Authors:** Chia-Wen Tsao

**Affiliations:** Department of Mechanical Engineering, National Central University, Taoyuan 32001, Taiwan; cwtsao@ncu.edu.tw; Tel.: +886-3-426-7343

**Keywords:** polymer microfluidics, polymer microfabrication, thermoplastics, polydimethylsiloxane

## Abstract

Using polymer materials to fabricate microfluidic devices provides simple, cost effective, and disposal advantages for both lab-on-a-chip (LOC) devices and micro total analysis systems (μTAS). Polydimethylsiloxane (PDMS) elastomer and thermoplastics are the two major polymer materials used in microfluidics. The fabrication of PDMS and thermoplastic microfluidic device can be categorized as front-end polymer microchannel fabrication and post-end microfluidic bonding procedures, respectively. PDMS and thermoplastic materials each have unique advantages and their use is indispensable in polymer microfluidics. Therefore, the proper selection of polymer microfabrication is necessary for the successful application of microfluidics. In this paper, we give a short overview of polymer microfabrication methods for microfluidics and discuss current challenges and future opportunities for research in polymer microfluidics fabrication. We summarize standard approaches, as well as state-of-art polymer microfluidic fabrication methods. Currently, the polymer microfluidic device is at the stage of technology transition from research labs to commercial production. Thus, critical consideration is also required with respect to the commercialization aspects of fabricating polymer microfluidics. This article provides easy-to-understand illustrations and targets to assist the research community in selecting proper polymer microfabrication strategies in microfluidics.

## 1. Introduction

With the introduction of microfluidics, micro total analysis system (μTAS), and lab-on-a-chip (LOC) devices in the 1900s, the use of microfluidic devices has increased tremendously due to the great potential in biomedical, point-of-care testing, and healthcare applications. The early development of microfluidic devices commonly involved silicon and glass materials as basic substrates. However, with the concept of using polymer materials in microfluidics been proposed in the late 1990s [1], the use of silicon and glass materials has shifted to polymers, primarily due to their simple and low-cost advantages. Compared to silicon and glass, polymers are inexpensive materials and feature a wide variety of material properties for meeting the various application requirements of disposable biomedical microfluidics devices, as well as many promising applications [2,3,4].

Fabrication of polymer microfluidic devices is relatively simple and no hazardous etching reagent is required to create the polymer microstructures. The fabrication tools for making polymer devices are also much cheaper than those for making semiconductor infrastructures, such as wet benches or reactive-ion etching facilities. These factors make it possible for polymer microfluidics devices to be easily fabricated in average research labs, a fact which has driven the development of polymer microfluidics academically, and further toward industrial applications. After years of polymer microfluidics investigations, various polymer microfabrication technologies have been developed using simple and low-cost formats. However, polymer microfabrication is not a straightforward process and, as yet, there is no one-fits-for-all fabrication technique for creating polymer microfluidic devices. Proper determination of polymer microfabrication strategies is critical for successful polymer microfluidic device functionality. In this paper, we examine polymer microfabrication with respect to the raw materials, facility costs, and general and state-of-art fabrication processes, as well as commercialization considerations.

## 2. Selection of Polymer Material and Microfabrication Processes Selection

**Figure 1:**
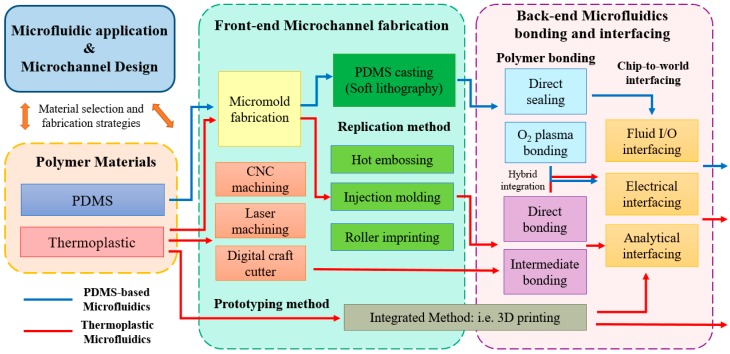
Polymer microfluidics fabrication process chart. The blue line indicates the PDMS-based microfluidics fabrication procedure, and the red line indicates the thermoplastic microfluidics fabrication procedure.

In the polymer microfabrication process, the first step is to identify its application and requirements. Once the microfluidic chip application is identified, the microchannel/chamber layouts can be designed. Next, one selects an appropriate polymer material and determines the fabrication strategy to create a polymer microfluidic device that will meet the specific microfluidic application requirements. The polymer materials typically used in microfluidic applications can be divided into two major categories: polydimethylsiloxane (PDMS) and thermoplastics. Figure 1 shows the polymer microfluidics fabrication procedures and selection strategies associated with PDMS (blue line) and thermoplastics (red line). PDMS is one of the major materials used in polymer microfluidics because of material elasticity, gas permittivity, and other several unique advantages. PDMS is an elastomer material that can be deformed under the application of force or air pressure. The PDMS valve was invented to control microchannel fluidic transportation, which also enables very large scale integration in high-throughput applications [5,6,7]. Both PDMS and thermoplastics materials have shown high biocompatibility for many biomolecules and cells [8,9]. Due to its high gas permittivity property and high optical transmissivity, PDMS is the main material choice for cell-based microfluidic devices [7,10,11]. Although PDMS has advantages, it also has several limitations in microfluidic applications. Problems, such as channel deformation, low solvent and acid/base resistivity, evaporation, sample absorption, leaching, and hydrophobic recovery, are the fundamental challenges associated with PDMS in microfluidic devices [12,13]. Thermoplastics are synthetic polymers that have various surface properties for microfluidic application. Thermoplastics such as poly(methyl methacrylate) (PMMA), polycarbonate (PC), polystyrene (PS), polyvinyl chloride (PVC), polyimide (PI), and the family of cyclic olefin polymers (i.e., cyclic olefin copolymer (COC), cyclic olefin polymer (COP), and cyclic block copolymer (CBC)) have been widely used in microfluidics. [14,15,16]. Thermoplastics are rigid polymer materials that have good mechanical stability, a low water-absorption percentage, and organic-solvent, and acid/base resistivity, which are critical factors in many bioanalytical microfluidic applications, such as high-pressure liquid chromatography (HPLC) microfluidic applications [17], that involve a high-pressure solvent injection procedure. PDMS may suffer from solvent swelling and channel deformation issues, which makes thermoplastics (like COC) an ideal choice for the polymer material. Table 1 summarizes the typical mechanical, optical, chemical (solvent and acid/base resistance), and material costs for PDMS and thermoplastics commonly used in microfluidics.

## 3. Polymer Microfluidics Fabrication Procedure

### 3.1. PDMS and Thermoplastic-Based Polymer Microfluidics

The fabrication process of PDMS microfluidic chips is relatively straightforward [18,19]. As shown in the blue process lines in Figure 1, the PDMS microchannel is mainly fabricated by a simple soft lithography process in which the PDMS reagent is directly cast onto a master micromold [20], followed by a bonding process [21]. The typical PDMS casting procedure is performed by mixing a PDMS base with a curing reagent in a 10:1 ratio, followed by curing at 80 °C for 1–2 h. The PDMS layer is then released from the micromold to complete the casting procedure. Since the casting process is such a simple process and the layer is easily released from the micromold, PDMS casting is a reliable and high yield procedure. SU-8 resin and standard photoresist (PR) can be used as micromolds in the PDMS procedure [22]. Sealing of the PDMS microstructure to enclose a microfluidic channel or chamber also involves a simple and reliable procedure. A PDMS layer can be directly sealed/stuck to another PDMS or glass substrate via van der Waals forces without the need for further fabrication procedures. To meet high bonding strength requirements, the PDMS bond strength can be enhanced by tuning the process parameter [21] or, more commonly, using oxygen plasma treatment to form an O–Si–O covalent bond at the PDMS interface [23,24].

In the thermoplastic microfluidic chip fabrication procedure (Figure 1, red lines), there are various fabrication options for making thermoplastic microchannels. Thermoplastic microchannels can be created either by rapid prototyping or replication methods. Rapid prototyping methods, such as computer numerical controlled (CNC) milling [25,26,27], and laser ablation [28,29] are available for generating microchannels on the thermoplastic substrate. Recently, a low-cost rapid prototyping method by a digital craft cutter was proposed to create microchannels on a thin transparent thermoplastic film [30,31,32]. Although CNC, laser ablation, or digital craft cutter methods have limits with respect to microchannel resolution and surface roughness, they are important procedures in thermoplastic microfluidics fabrication because rapid prototyping is a simple process for researchers to establish proof-of-concept without the need for micromold fabrication. For mass production, thermoplastic microchannels can be fabricated by replication processes, such as hot embossing/imprinting [33,34,35], roller imprinting [36,37], and injection molding [38,39], which are common polymer replication methods for massively reproducing thermoplastic microchips. In the thermoplastic fabrication process, bonding is a critical last step that determines the bonding strength, geometry stability, optical transmissivity, and surface chemistry of the produced microfluidic device. In some bonding processes, there can be bottlenecks in the mass production of thermoplastic microfluidic devices. Issues associated with bonding throughput are detailed in Section Section 4. A comprehensive review of thermoplastic bonding methods have been reported by Tsao and DeVoe [40]. Generally, thermoplastic bonding is achieved either by direct bonding or an intermediate bonding approach. Direct bonding is a bonding process that uses no intermediate material at the bonding interface. Methods such as thermal fusion bonding [41,42], ultrasonic welding [43], surface modification [44,45,46], and solvent bonding [47,48] are categorized as direct bonding methods. Indirect bonding is defined as bonding that involves the use of an additional material or chemical reagents to assist in the bonding, such as epoxy, adhesive tape, or chemical reagents. Indirect thermoplastic bonding methods, such as adhesive bonding [49,50,51] or microwave bonding [52], use an intermediate layer, such as metal or a chemical reagent.

**Figure 2:**
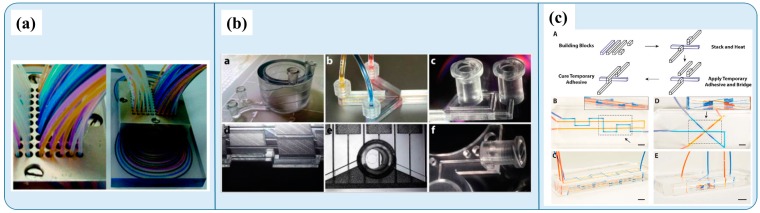
(**a**) Custom-designed chip-to-world multichannel interfacing. Reproduced from [63] with permission of The Royal Society of Chemistry; (**b**) polymer microfluidics device fabricated by 3D printing process. Reproduced from [78] with permission of The Royal Society of Chemistry; and (**c**) thermoplastic building blocks for versatile PDMS microfluidics. Reproduced from [74] with permission of The Royal Society of Chemistry.

After completing the microchannel fabrication and bonding process to seal the microchannels, the last step is to connect the microfluidic device for chip-to-world interface. Surface modification procedures are sometimes applied in the polymer microfluidics to meet specific application requirements [53,54,55,56,57,58,59]. Microfluidics interfacing issues remain a challenge and have been given less emphasis in the microfluidic community. A good interface is a critical aspect that determines the success of practical applications and commercialization potential. A recent review by Temiz et al. summarized methods on how to “plug” chips for post-end fluidics, electronics, and analytical interfaces [60]. Both PDMS and thermoplastic microfluidics chips commonly use a standard Luer lock/cone, or peek connector [61] for the fluidic interface. Solutions, such as surgical needles [62] or customer-designed connectors [63] (Figure 2a), for the fluidic inlet/outlet have also been proposed. In particular, due to the thermoplastic substrate’s rigidity, needles can achieve a tight-fit insertion into the thermoplastic in high-pressure fluidic connections, which makes thermoplastics an appealing material for high-pressure applications. For microfluidic devices to provide control and detection functions, based on electrical principles (i.e., electrophoresis, electrowetting, electrochemical sensing), electrode pads for power and electrical connections are required. The use of stainless surgical needles [62] in fluidic connection has demonstrated good electrical contact or power connection for electrophoresis or isoelectric focusing applications [64]. For integrated on-chip electrode pads, electrical contacts can be deposited on the polymer surface by thermal or electron beam [64,65], screen printing [66], or 3D ion implantation electrode [67]. Many microfluidic devices are analyzed by optical detection. Since all polymer materials are optically transparent (Table 1), optical detection can be directly performed on a microscope without any additional interface setup on the chip. However, for other detection methods, an analytical interface is required. For example, when interfacing with mass spectrometry analysis, an electrospray or droplet deposition orifice must be incorporated into the microfluidic device [68].

### 3.2. Advances of Polymer Microfluidics Fabrication

Today, polymer microfluidics continues to be an intriguing research topic. Various polymer materials have been demonstrated in the microfluidic applications with better performance [69]. For example, thermoset polyester (TPE) was proposed in microfluidics as an alternative material to PDMS providing better chemical and solvent compatibility. The TPE fabrication process is also compatible with standard replica molding, as well as advanced rapid high-pressure injection procedures [70,71,72]. In polymer microfluidics fabrication, there is no one-fit-for-all polymer fabrication technique and research is ongoing to identify techniques that are more reliable, simple, versatile, and robust. In polymer replication, Beebe et al. recently reported a thermoplastic bonding method that combines hot embossing and milling for faster replication, based on the hot embossing method [73]. Several novel micromold technologies have been developed to realize better polymer replication performance. For example, the thermoplastic building blocks technique (Figure 2c) offers micromold design flexibility for producing PDMS microfluidic devices for diverse geometries and functionalities [74]. Liquid metal alloys (bulk metallic glass) can also be integrated into microfluidic molding technology to achieve more robust and versatile polymer fabrication [75,76]. Recently, 3D printing technologies [77,78] (Figure 2b) have become a popular prototyping method for fabricating the polymer microfluidic devices.

With respect to the post-end microfluidic bonding advances, reversible bonding, based on re-melting the wax [79] or a magnetic force [80] enables the production of dismountable and reusable microfluidic devices. Bonding a heterogeneous material to make a “hybrid” device is also an important method for making advanced integrated microfluidic devices. Bonding PDMS with thermoplastic material enables a wider range of microfluidic applications. Tan et al. introduced a PMMA–PDMS pneumatic micropump as a hybrid microfluidic device using optically-clear adhesive film [81]. Li et al. used a selective stamp bonding technique to transfer epoxy to bond a PDMS–polystyrene (PS)/poly(ethylene terephthalate) (PET) microfluidic device for human lung epithelial cells analysis [82]. A doubly cross-linked nano-adhesive method has also been reported for sealing PDMS with polyimide (PI) or polyethylene terephthalate (PET) [83]. Bonding polymer with paper can integrate thermoplastic material with novel microfluidic paper-based analytical devices (µPADs) [84].

## 4. Commercialization Considerations for Polymer Microfluidics Fabrication

Since publication of the first polymer microfluidic paper [85], the idea of using polymer material in microfluidics has become increasingly popular in the research community. With almost 20 years of development, polymer microfluidic technology has become the major material choice for microfluidics due to its advantages of low cost and disposability, and many effective bioanalytical applications have been demonstrated. Microfluidic devices are currently in the technology transfer stage from the research lab to commercial production. PDMS and thermoplastics each have their own advantages for microfluidics applications, which are also indispensable factors in choosing materials for commercialized products. For example, Fluidigm Inc.’s (South San Francisco, CA, USA) integrated fluidic circuits are generated using a PDMS soft lithography process and the HPLC chips from Agilent Technologies (Santa Clara, CA, USA) are based on a PMMA thermoplastic substrate. Several microfabrication foundries, such as MiniFAB or Micralyne, have provided a fabrication-services business model for the mass fabrication of polymer microfluidic devices. Many emerging microfluidic devices are also currently being transferred from research prototypes into products. In general, a low-volume (<200 pieces per month) polymer production rate is appropriate for academic or research labs developing prototypes for proof-of-concepts. For commercialized microfluidic devices, medium volume (200–2000 pieces per month) or preferably high-volume (>2000 piece month), mass production strategies should be considered. In addition to the material properties and performance of polymer microfluidic devices, fabrication throughput is a particularly important consideration for the commercialization of polymer microfluidic devices.

**Figure 3:**
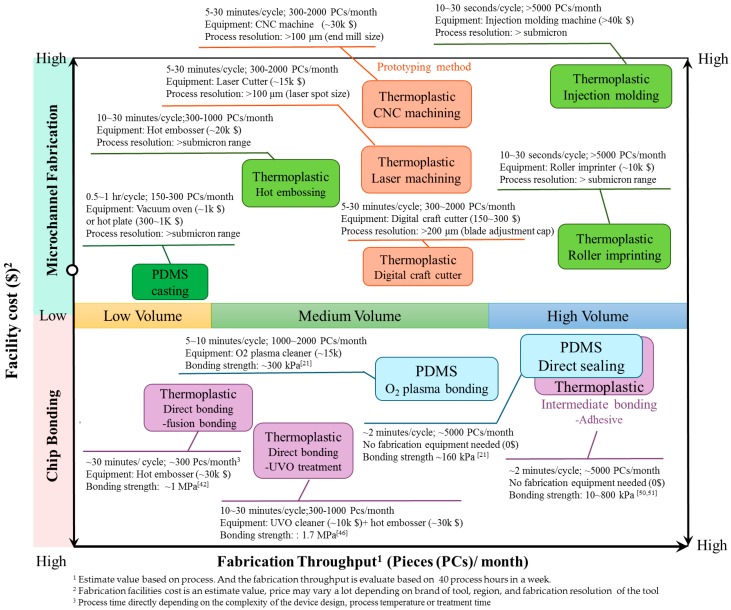
Estimation of fabrication throughput (*x*-axis, PCs/month) and facility cost (*y*-axis, in US dollars) of critical polymer microfabrication procedures.

Figure 3 shows the key polymer microfluidic fabrication procedures (microchannel fabrication and chip bonding) in terms of their facility cost and fabrication throughput. From the fabrication perspective, the PDMS casting process is time-consuming, normally taking 0.5–1 h to complete a casting cycle, and can thus provide only 150–300 devices per month at a standard research-lab scale. This may potentially constrain the production of high-volume quantities of PDMS microfluidic devices. With respect to PDMS bonding, because PDMS can be directly sealed to the glass or PDMS layer, bonding can be achieved by a simple attachment procedure without the need for any bonding facility. Even for high bond strength O_2_ plasma bonding, PDMS bonding can be achieved within 10 min using O_2_ plasma activation. By combining the PDMS casting and sealing procedures, PDMS microfluidic devices can achieve medium-volume fabrication. Additoinally, PDMS chip fabrication facilities can be developed in low-budget conditions (i.e., hot plate/vacuum oven and plasma cleaner) while achieving good microfluidic throughput for research investigations. As such, PDMS has sometimes been a more popular microfluidic chip choice than thermoplastics in academic research labs. 

With respect to thermoplastics, because there is more variety of choice in the fabrication process, thermoplastic microdevices can be generated either by low–medium throughput prototyping/replication or by high-throughput replication methods. For the commonly used hot embossing process, depending on the heating/cooling conditions, microchannels can be replicated in a medium-volume production range at a rate of 10–30 min/cycle. In particular, methods such as injection molding and the continuous reel-to-reel roller imprinting method can be complete a replication cycle within seconds, which is ideal for producing large numbers of replicas per day, as required for commercial manufacture. Regarding the thermoplastic bonding process, a wide variety of thermoplastic bonding methods have been reviewed previously [40]. For comparison with other fabrication methods, in Figure 3, we show three commonly used bonding methods: thermal fusion bonding, surface treatment (UV/ozone, UVO), and adhesive bonding. In the direct fusion bonding method, because it requires thermoplastic heating above T_g_ to “fuse” the bonding pairs, a longer time of around 30 min/cycle is required to bond a chip. A surface treatment bonding method has been proposed to effectively reduce the processing temperature below T_g_ or even to room temperature. Depending on the bonding temperature, the process cycle time can be reduced to ~10 min/cycle for the evaluated production volume. Using adhesive bonding, the thermoplastic can be bonded at room temperature, so chips can be bonded rapidly within 2 min to achieve high-volume polymer fabrication. We note that the facilities costs and fabrication throughputs in Figure 3 are estimated values, and the price and processing times may vary depending on the tool brand and the fabrication resolution. Nevertheless, Figure 3 provides a useful comparison of the fabrication throughput and cost aspects. With the selection of the more appropriate fabrication method, both PDMS and thermoplastic materials can reach medium- to high-volume fabrication throughput to meet mass commercial production requirements.

## 5. Conclusions

PDMS and thermoplastics are two important substrate materials in polymer microfluidics and polymer microfabrication techniques that have been well developed for both to begin transferring microfluidics prototypes from academic research labs to commercialized production. The proper selection of polymer material and polymer microfabrication strategy are critical to ensure success in polymer microfluidics research and commercialization. Future development of polymer microfabrication techniques should further explore the microfabrication performance (i.e., minimum channel resolution, bonding strength, etc.), but also consider commercial aspects (i.e., fabrication throughput and cost) to bridge polymer microfluidic devices from research prototypes into commercialized products.

## Figures and Tables

**Table 1:** Summary of physical properties and suppliers for common polymer microfluidic materials.

Polymer		PDMS	Thermoplastics			
			PC	PMMA	PS	COC/COP/CBC
Mechanical property		Elastomer	Rigid	Rigid	Rigid	Rigid
Thermal property ^1^		~80 °C	140~150 °C	100~125 °C	90~100 °C	70~155 °C
Solvent resistance		Poor	Good	Good	Poor	Excellent
Acid/base resistance		Poor	Good	Good	Good	Good
Optical transmissivity	Visible range	Excellent	Excellent	Excellent	Excellent	Excellent
	UV range	Good	Poor	Good	Poor	Excellent
Biocompatibility		Good	Good	Good	Good	Good
Material cost ^2^		~150 $/Kit (1 Kg) ^3^	<3 $/Kg ^3^	2~4 $/Kg	<3 $/Kg	20~25 $/Kg ^4^

^1^ Thermal property is determined based on the PDMS curing temperature and thermoplastic glass transition (T_g_) temperature; ^2^ The cost information is provided by a local supplier. Cost may be different in different regions. Thermoplastic material is in pellets; ^3^ Suppliers: Dow Corning, Midland, MI, USA; ^4^ Suppliers: JSR ARTON (Tokyo, Japan), ZEON Chemicals (Louisville, KY, USA), TOPAS Advanced Polymers (Florence, KY, USA), USI Corporation (Taipei, Taiwan).
